# Intelligence

**Published:** 1828-12

**Authors:** 


### INTELLIGENCE.

### MONTHLY REPORT OF PREVALENT DISEASES.

The diseases prevalent during the past month have, like the state of the weather, resembled those of summer rather than winter. We have met with several instances of rather severe diarrhœa, in which considerable quantities of bile have been passed. They have generally done well under the use of the Hydrargyrum cum Creta, with Dover's powder.—Fever has also been rather frequent, and some cases of it have proved obstinate, though in general the character of the disease has been mild.

Our readers will probably remember the notice we gave of Mr. Marshall's "Hints to Medical Officers." The followiog extract from the Army List for

November affords a proof that we did not overrate the favorable impression that the work would create.

*Circular addressed to the Medical Officers of Regiments.*

*Army Medical Department.*

A late publication of Staff-Surgeon Henry Marshall, intituled "Hints to young Medical Officers of the Army, on the Examination of Recruits," &c. printed by Burgess and Hill, contains information and instructions which, if duly attended to, will much assist army medical officers of all ranks, especially the younger classes, in discharging this important duty. I accordingly strongly recommend the purchase of this volume; and have—, &c.

J. M'GRIGOR, *Director General.*

---

*Regulations of the Court of Examiners of the Society of Apothecaries.*—Mr. Watson, secretary to the Court of Examiners at Apothecaries' Hall, has published, in the Medical Gazette, the following explanation of the regulations of 1826, 7, and 8:

All medical students who commenced their attendance on lectures prior to the 1st of February, 1828, will be admitted to be examined agreeably to the regulations of 1826: viz. after an attendance on one course of lectures on chemistry; one course of lectures on materia medica; two courses of lectures on anatomy and physiology; two courses of lectures on the theory and practice of medicine; and six months physician's practice at an hospital, or nine months at a dispensary.

Those who *began* to attend lectures subsequently to the 1st of February, 1828, and previously to the present month (October), will be expected to comply with the regulations of 1827, and will only be admitted to be examined after the following course of study: viz. an attendance on one course of lectures on chemistry; one course of lectures on materia medica and medical botany; two courses of lectures on anatomy and physiology; two courses of lectures on the theory and practice of medicine; (these last to be attended subsequently to the lectures on chemistry and materia medica, and to one course, at least, of anatomy;) and six months, at least, physician's practice at an hospital, or nine months at a dispensary; (such attendance to commence subsequently to the termination of the first course of lectures on the principles and practice of medicine.)

Those students whose attendance on lectures commenced in the present month, will be required to observe the regulations of 1828: viz. to attend two courses of lectures on chemistry; two courses of lectures on materia medica and botany; two courses of lectures on anatomy and physiology; two courses of anatomical demonstrations; two courses of lectures on the theory and practice of medicine; (these last to be attended subsequently to one course of lectures on chemistry, materia medica, and anatomy;) and six months, at least, physician's practice at a hospital, or nine months at a dispensary; (such attendance to commence subsequently to the termination of the first course of lectures on the principles and practice of medicine.)

But all students who shall commence their attendance on lectures at the second course of the present winter session, (namely, in January 1829,) will be required to attend the physician's practice at an hospital for nine months, or at a dispensary for twelve months.

*Medico-Chirurgical Society.*—The most interesting paper read during the present season has been one on *Inflammation of the Veins*, by Mr. ARNOTT, an account of which we subjoin, from the Medical Gazette.

After remarking upon the obscurity involving the symptoms attendant upon inflammation of veins, as well as the difficulty of accounting for the formation of matter in distant parts, occasionally following injuries, Mr. Arnott stated that, in three cases of inflammation of veins which had fallen under his observation, he found in one a deposition or pus, without any sign of previous inflammation, under the skin of the forearm on the opposite side; in another, destructive inflammation of the knee-joint, with pus in the cellular texture of the thigh; while in none of the three did the inflammation of the vein extend to the heart. These cases led him to examine the opinions advanced by different writers of repute upon this subject, and the doctrines of Mr. HUNTER, Mr. ABERNETHY, Mr. HODGSON, Mr. TRAVERS, Mr. CARMICHAEL, MM. BRESCHET, RIBES, &c. were severally adverted to; the result of which examination was, that even those explanations of the phenomena which possess most verisimilitude rest on uncertain grounds; a circumstance which Mr. Arnott thinks attributable rather to the subject not having received sufficient consideration, than to the absence of sufficient data on which to form correct opinions. In conformity with this view, he proceeded to detail succinctly a number of cases where death had resulted from phlebitis, and drew various conclusions from these. The first was, that there is no evidence of the inflammation of the vein extending to the heart. In ten cases which resulted from venesection, the vena cava was not affected, still less the heart; and in half of them the inflammation had not even extended to the axillary vein; and as the cases sometimes prove fatal where but a small portion only is inflamed, it would appear that there is no direct relation between the degree of danger and the extent of vein inflamed.

The next question is, whether the secondary affection depends upon pus entering into the circulation. On referring, for this purpose, to the cases on record, Mr. Arnott found that, in fourteen out of seventeen cases, pus, either alone or with lymph, was found in the vessel after death: in one case only was neither pus nor lymph found. From this it would appear probable that the entrance of the pus into the circulation is a principal, but not the sole, cause of the secondary affection. The early appearance of the symptoms in some cases is scarcely compatible with the time required for the formation of pus, and therefore it is most likely that, if the secondary effects result from the passage of any fluid into the blood, it is of inflammatory secretions generally, and not of pus alone. According to the observations of Mr. Arnott, the inflammation of the vein usually terminates where some other vessel joins that which is inflamed. He first noticed this in a horse which was affected with phlebitis from bleeding, and in which the inflammation of the jugular suddenly stopped at the point where a small vein entered it. Soon after, in examining the body of a man who had had phlebitis, he found the inflammation of the femoral vein extending along the external iliac, to the point where the internal iliac joined it; and in a case of inflammation of the left spermatic vein, the diseased appearances extended through the emulgent vein, but ceased abruptly where this entered the cava. Mr. A. went on to shew that facts in confirmation of this general idea had been incidentally mentioned by several of those who have recorded cases of phlebitis.

The author next described the symptoms of phlebitis, and stated the periods at which death took place in a certain number of recorded cases. On examining the bodies of those who die, the following are the appearances which most frequently present themselves:—Effusions into the chest of a sero-purulent character, and the general sequelæ of active inflammation; but especially purulent depositions, either infiltrated or as distinct abscesses. The same appearances occasionally manifest themselves in the cellular substance of different parts of the body, or in some of the parts of the eye; in some instances these phenomena have been found within the cranium. The disease of the joints, in one case which was detailed, consisted of violent inflammation of the synovial membrane, with ulceration of the cartilages and baring of the bones.

Mr. Arnott pointed out the great resemblance between the train of symptoms marking the secondary symptoms in phlebitis and those which arise from the inoculation of poisons. There is in both a local affection, which is frequently very inconsiderable; and to this succeeds great constitutional disturbance, followed by inflammation of a peculiar and severe character in different parts of the body. The resemblance, which in a general point of view is sufficiently obvious, is nevertheless particularly striking with regard to the phenomena attending wounds received in dissection. There are in both a train of symptoms nearly similar, succeeded by the development of inflammation at distant points, and this also attacking nearly similar parts in both. Mr. Arnott illustrated this by a reference to several cases of death from injury received in dissection.

The fact that purulent matter is sometimes found without any signs of previous inflammation has been long known, and has been called abscess by metastasis, it having been imagined that the pus was taken up and deposited ready formed in some other place. Mr. Cheston, in his Pathological Observations, (1766,) particularly alludes to this pheuomenon, and expressly says that the matter is rather disseminated through the viscus than collected into an abscess. Mr. Hunter denied the possibility of purulent matter being translated from one part to another; but it was maintained in Italy by Monteggia, who describes the serous membranes of the great cavities as particularly obnoxious to the action of absorbed matters, which, he adds, also produce abscess in particular viscera, especially the liver and lungs. More recently attention has been directed to the subject by Mr. Guthrie, Mr. Bell, M. Velpeau, and Mr. Rose.

Mr. Arnott argues that, as all the evils above enumerated have been known to follow the puncture, division, or ligature of a vein, it is probable that, when they have succeeded to a more extensive injury, they have still in reality owed their origin to the same cause, namely, inflammation of one or more veins. But, to confirm this, we ought, on the one hand, to find inflammation of the veins where the consequences alluded to have followed injuries; and, on the other hand, we ought to find similar secondary consequences under circumstances in which it is known that inflammation of veins is a frequent pathological condition, as after parturition. Mr. A. then proceeded to shew that such was the case. He first detailed four instances in which secondary affections of the viscera occurred after injuries of the extremities, complicated with inflammation of the veins of the wounded limb. In injuries of the head, secondary affections of the viscera of the chest and abdomen have long been observed; and Desault, who has particularly noticed the formation of abscess in the liver under such circumstances, attributed the phenomena to concussion of the brain; an idea adopted by others, but which was founded merely on conjecture.

The author of the paper here referred to thirty-two cases in which affections of the thoracic and abdominal viscera succeeded to injuries of the head. The secretary did not read these, but proceeded to the general summary, which was, that the injury of the head in these cases consisted in twenty-two of fracture, which in all was compound, (except one, with regard to which the circumstance is not stated;) in ten there was no fracture; but in every instance there was wound of the soft parts. The wound of the soft parts was the only circumstance common to all the thirty-two cases. The phenomena attending the formation of these visceral affections were so similar to those succeeding wounds of other parts, that Mr. A. thinks it fair to attribute them to the same cause.

Mr. Arnott next proceeded to remark, that inflammation of the veins was common after parturition, and quoted several cases to shew that there was visceral affection under such circumstances, although it had not been much attended to. He next adverted to the affection of the joints, and mentioned several cases in which it had been distinctly connected with inflammation of the veins, particularly in a patient who died a short time ago, in Middlesex Hospital, with disease of the left knee and right shoulder joints, and collections of matter over the scapula and sacrum; and in which the author, in consequence of the similarity to other cases, anticipated the existence of inflamed veins, and confirmed his opinion by examining the limb, when he found the femoral vein in a state of inflammation; a preparation of which was exhibited. The author next spoke of a severe affection of the joints as occurring in parturient women, and mentions various authorities in corroboration; detailing at length an interesting case communicated to him by Dr. Lee.

In order to extend the analogy, and endeavouring to draw the connexion between these cases still closer, Mr. Arnott next alluded to the occurrence in the parturient state of a disease of the eye, similar to that which had occurred in two cases of phlebitis: one treated by Mr. Earle, and the other a patient in whom Mr. Wardrop had tied the carotid artery with the effect of obliterating the jugular vein. This disease of the eye after delivery, it will be remembered, was made the subject of a paper published by Dr. M. Hall and Mr. Higginbottom, in the Transactions of the Medical Society, about two or three years ago.

The general conclusions at which Mr. Arnott arrived at the termination of his paper was, that the abscesses and inflammations which take place in remote situations, after injuries of the extremities, of the head, or after parturition, are dependent upon the existence of phlebitis in the part originally affected. He does not regard the diseased action as one consisting in a mere metastasis, or change of situation in absorbed matter; but that the secondary local affections derive their peculiar characters from a change induced in the blood by its admixture with the pus, or other inflammatory secretions from the vein.

### METEOROLOGICAL JOURNAL,

*From October 20th, to November 20th,* 1828.

By Messrs. HARRIS and Co. Mathematical Instrument Makers, 50, High Holborn.

| October | Rain gauge. | Moon. | Thermom. | | | Barometer. | | De Luc's Hygrom. | | Winds. | | Atmospheric Variations. | | |
|---|---|---|---|---|---|---|---|---|---|---|---|---|---|---|
| | | | 9 A.M. | MAX. | MIN. | 9 A.M. | 10 P.M. | 9 A.M. | 10 P.M. | 9 A.M. | 10 P.M. | *9 a.m.* | *2 p.m.* | *10 p.m.* |
| 20 | | | 40 | 53 | 45 | 29.99 | 29.99 | 53 | 54 | E | E | Fine | Fine | Fine |
| 21 | | | 52 | 58 | 46 | .99 | .94 | 56 | 65 | E | N | Fog | Fine | Fog |
| 22 | | | 55 | 63 | 54 | .80 | .69 | 52 | 63 | E | WSW | Fine | Fine | Fine |
| 23 | | ○ | 58 | 59 | 42 | .60 | .78 | 64 | 65 | WSW | NNE | Rain | Rain | Cloudy |
| 24 | .16 | | 44 | 52 | 38 | 30.01 | 30.04 | 64 | 60 | N | NW | Fine | Fine | Fine |
| 25 | | | 42 | 55 | 43 | .07 | .15 | 61 | 55 | W | SSW | Foggy | Fine | Fine |
| 26 | | | 48 | 52 | 48 | .11 | .08 | 61 | 65 | SSW | SSW | —— | Rain | Sl. Rain |
| 27 | | | 50 | 53 | 50 | .06 | .16 | 65 | 66 | SE | SE | Rain | Rain | Rain |
| 28 | .21 | | 51 | 54 | 40 | .28 | .32 | 68 | 65 | ESE | E | Cloudy | Fine | Fine |
| 29 | | | 46 | 53 | 42 | .34 | .24 | 64 | 58 | E | E | Fine | Fine | Fine |
| 30 | | ☾ | 45 | 52 | 39 | .17 | .18 | 55 | 58 | E | E | —— | —— | Sl. Fog |
| 31 | | | 42 | 51 | 45 | .14 | .15 | 60 | 60 | ESE | ENE | Sl. Fog | Fine | Cloudy |
| Nov. | | | | | | | | | | | | | | |
| 1 | | | 47 | 52 | 46 | .14 | .11 | 60 | 58 | ENE | NNE | Foggy | Fine | Cloudy |
| 2 | | | 48 | 53 | 45 | .10 | .16 | 56 | 58 | N | NNE | Fine | Fine | Fine |
| 3 | | | 46 | 52 | 42 | .20 | .20 | 60 | 60 | NNE | ENE | Foggy | Fine | Fine |
| 4 | | | 45 | 53 | 40 | .14 | .07 | 62 | 62 | ENE | SE | —— | —— | Foggy |
| 5 | | | 42 | 53 | 40 | .04 | 29.99 | 63 | 63 | SE | ESE | Fine | Fine | Cloudy |
| 6 | | | 45 | 53 | 40 | 29.99 | .96 | 65 | 66 | SSE | SSE | Foggy | Foggy | Foggy |
| 7 | | ● | 46 | 46 | 35 | .90 | .81 | 67 | 60 | SE | SE | Fine | Fine | Fine |
| 8 | | | 37 | 45 | 34 | .80 | .77 | 55 | 53 | ESE | ESE | —— | —— | —— |
| 9 | | | 36 | 44 | 36 | .60 | .50 | 55 | 57 | ESE | ESE | —— | —— | Foggy |
| 10 | | | 39 | 38 | 34 | .40 | .38 | 60 | 63 | ESE | ESE | Foggy | Fine | Cloudy |
| 11 | | | 36 | 34 | 26 | .44 | .50 | 65 | 64 | E | E | —— | Foggy | Foggy |
| 12 | | | 28 | 34 | 28 | .51 | .51 | 64 | 66 | ENE | ENE | —— | Fog | Fog |
| 13 | | | 38 | 46 | 45 | .55 | .56 | 68 | 70 | E | SW | Fine | Fine | Fine |
| 14 | | ☽ | 48 | 45 | 40 | .39 | .24 | 71 | 74 | SE | S | Foggy | Rain | Cloudy |
| 15 | .40 | | 45 | 54 | 48 | .21 | .24 | 75 | 72 | SSE | SW | Rain | Cloudy | Cloudy |
| 16 | | | 44 | 54 | 47 | .20 | .18 | 72 | 70 | SW | W | Rain | Rain | Rain |
| 17 | .43 | | 50 | 56 | 44 | .50 | .66 | 70 | 72 | NNW | WNW | Fine | Cloudy | Fine |
| 18 | | | 45 | 50 | 41 | .80 | .92 | 74 | 68 | WNW | NW | Foggy | Fine | Fine |
| 19 | | | 45 | 50 | 40 | .81 | 30.06 | 66 | 65 | NW | NW | Fine | —— | —— |

The quantity of Rain fallen in the month of October, was — inch and 52.100ths.

